# Modeling the population‐level impact of opioid agonist treatment on mortality among people accessing treatment between 2001 and 2020 in New South Wales, Australia

**DOI:** 10.1111/add.15736

**Published:** 2021-12-04

**Authors:** Antoine Chaillon, Chrianna Bharat, Jack Stone, Nicola Jones, Louisa Degenhardt, Sarah Larney, Michael Farrell, Peter Vickerman, Matthew Hickman, Natasha K. Martin, Annick Bórquez

**Affiliations:** ^1^ Division of Infectious Diseases and Global Public Health University of California San Diego CA USA; ^2^ National Drug and Alcohol Research Centre University of New South Wales Randwick NSW Australia; ^3^ Population Health Sciences University of Bristol Bristol UK; ^4^ Centre de Recherche du Centre Hospitalier de l'Université de Montréal (CRCHUM) and Department of Family Medicine and Emergency Medicine, Université de Montréal Montréal Canada

**Keywords:** Incarceration, mathematical modeling, mortality, opioid agonist treatment, opioid use disorders, overdose

## Abstract

**Background and Aims:**

The individual‐level effectiveness of opioid agonist treatment (OAT) in reducing mortality is well established, but there is less evidence on population‐level benefits. We use modeling informed with linked data from the OAT program in New South Wales (NSW), Australia, to estimate the impact of OAT provision in the community and prisons on mortality and the impact of eliminating excess mortality during OAT initiation/discontinuation.

**Design:**

Dynamic modeling.

**Setting and participants:**

A cohort of 49 359 individuals who ever received OAT in NSW from 2001 to 2018.

**Measurements:**

Receipt of OAT was represented through five stages: (i) first month on OAT, (ii) short (1–9 months) and (iii) longer (9+ months) duration on OAT, (iv) first month following OAT discontinuation and (v) rest of time following OAT discontinuation. Incarceration was represented as four strata: (i) never or not incarcerated in the past year, (ii) currently incarcerated, (iii) released from prison within the past month and (iv) released from prison 1–12 months ago. The model incorporated elevated mortality post‐release from prison and OAT impact on reducing mortality and incarceration.

**Findings:**

Among the cohort, mortality was 0.9 per 100 person‐years, OAT coverage and retention remained high (> 50%, 1.74 years/episode). During 2001–20, we estimate that OAT provision reduced overdose and other cause mortality among the cohort by 52.8% [95% credible interval (CrI) = 49.4–56.9%] and 26.6% (95% CrI =22.1–30.5%), respectively. We estimate 1.2 deaths averted and 9.7 life‐years gained per 100 person‐years on OAT. Prison OAT with post‐release OAT‐linkage accounted for 12.4% (95% CrI = 11.5–13.5%) of all deaths averted by the OAT program, primarily through preventing deaths in the first month post‐release. Preventing elevated mortality during OAT initiation and discontinuation could have averted up to 1.4% (95% CrI =  0.8–2.0%) and 3.0% (95% CrI = 2.1–5.3%) of deaths, respectively.

**Conclusion:**

The community and prison opioid agonist treatment program in New South Wales, Australia appears to have substantially reduced population‐level overdose and all‐cause mortality in the past 20 years, partially due to high retention.

## INTRODUCTION

World‐wide, nearly 500 000 deaths in 2019 were directly related to drug use, of which nearly 100 000 were caused by opioid use disorders (OUD) [1, 2]. Opioid‐related deaths have continued to steeply rise over the COVID‐10 pandemic and represent a public health crisis in many countries [3, 4].

Opioid agonist treatment (OAT), with medications such as methadone and buprenorphine, is an evidence‐based treatment which individual‐level analyses have shown reduces mortality due to opioid overdose and other causes [5, 6] and prevents acquisition of HIV [7] and hepatitis C virus [8] among people who use opioids, especially if they inject drugs [9]. As a result, numerous public health agencies, such as the World Health Organization, recommend the provision of OAT [10, 11, 12, 13]; methadone and buprenorphine are listed as WHO essential medicines for this indication.

While the individual‐level efficacy of OAT on reducing mortality has been established and quantified in meta‐analyses [5, 6], demonstrating its effect at population‐level has been more challenging. Such evidence is key to support and inform scale‐up of OAT programs globally, which is urgently needed, as most programs reach only a small proportion of those in need [14]. Very few studies (mainly ecological) [15, 16, 17, 18, 19] have evaluated the impact of OAT provision on population‐level mortality, with conflicting results. Ecological studies in Europe found that the number of OAT recipients were inversely associated with the number of overdose deaths [15, 17]. In France, where since 1995 all registered physicians are allowed to prescribe bubprenorphie without speciality certification, the number of patients on OAT increased by more than 95% from 1995 to 1999 (from fewer than 2000 to more than 65 000 per year), and opiate‐related overdose deaths declined by 79% during this period [17]. A similar ecological study in Baltimore found no association between number on OAT and number of heroin‐related deaths from 1995 to 1999 (when purity of heroin was rising) but a significant inverse association from 2003 to 2009, which coincided with the scale‐up of buprenorphine (a 13‐fold increase during this period, while the number of overdose deaths decreased by nearly twofold [16]). In the United Kingdom, a cohort study found a low probability of impact of OAT on population mortality, suggesting that the observed duration of OAT is too short to achieve population benefit and reduce the number of overdose deaths [19]. In contrast to the statistical analyses above, dynamic modeling can be used to investigate the impact of OAT programs at the population level through mechanistically reproducing mortality dynamics through time [9, 20]. Capturing the processes underlying mortality patterns as a function of OAT engagement and other key factors modulating mortality risk, such as incarceration, provides us with a solid framework to both retrospectively evaluate and predict OAT program impact. To date, dynamic modeling has not been used to evaluate historic OAT program impact, which is key to producing rigorous evidence of its population‐level effectiveness and on the factors driving it needed to informing policy.

In the Australian state of New South Wales (NSW), OAT was scaled‐up in the 1980s and has achieved a high coverage, with more than 40% of people who inject opioids reporting current use [21, 22, 23] (no estimates available among all people using opioids), thus providing a rich data source for understanding long‐term impact at population‐level. Additionally, OAT program data have been linked to public data sets on mortality and incarceration, thereby allowing a more comprehensive investigation of the effect of different life events (e.g. incarceration, hospitalization) on OAT engagement and the effectiveness of OAT on the risk of overdose and other cause mortality [24].

In this analysis, we use dynamic modeling to estimate the population‐level impact of the NSW OAT program on opioid overdose and other cause mortality among those who ever received OAT from 2001 to 2020, incorporating OAT engagement patterns and incarceration effects on mortality. We also estimate the specific contribution of the prison OAT program to reducing mortality among this cohort and investigate the potential increased efficiency of the program if higher mortality on initiation and discontinuation of OAT was eliminated.

## METHODS

We designed a simulation model of fatal overdose and other cause mortality among people who ever received OAT in NSW. The model tracks changes in opioid use, OAT engagement and incarceration, incorporating heterogeneity in overdose and other mortality risks during these periods. Data from the literature and the NSW OAT cohort were used to inform the model's parameters. The model was calibrated using Bayesian methods to time‐series data on OAT engagement, incarceration and mortality patterns from the NSW OAT cohort to reproduce the observed dynamics and to both quantify and more clearly understand the impact of the OAT program. Full detail on the model assumptions and data used are provided in the Supporting information, but key information is provided below. The analysis was not pre‐registered and the results should therefore be considered exploratory.

### Model description

We developed a dynamic, deterministic mathematical model of fatal overdose and other cause mortality among people who ever received OAT in NSW from 2001 to 2020, incorporating incarceration patterns and OAT receipt. While the NSW OAT program started systematically collecting data in 1985 [25] we chose 2001 as our start date, as linked‐incarceration data are available from 2000 and the model incorporated past‐year incarceration.

**FIGURE 1:**
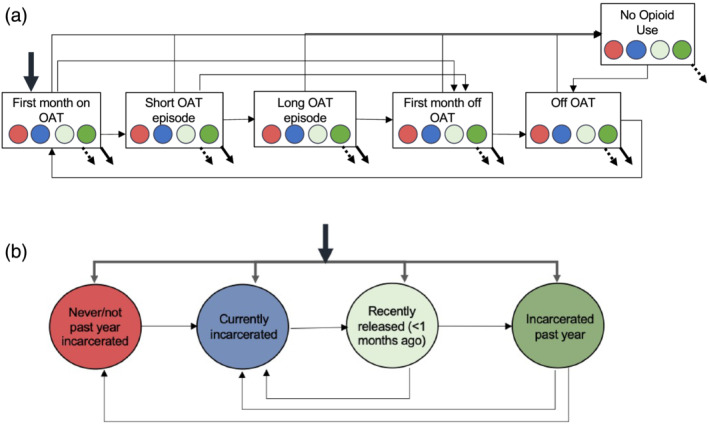
Model diagram of opioid agonist treatment (OAT), incarceration and associated overdose and other causes of death among the cohort of people who received OAT in NSW between 2001 and 2020. (a) Transitions between opioid use stages. Thin arrows represent progression through opioid use stages, including OAT and opioid use cessation; the thick arrow represents entry into the model (i.e. into the OAT cohort); dashed and full arrows at the bottom of each box represent death from other causes and deaths from overdose, respectively. Circles are colored by prison status (see panel B). (b) Transitions between incarceration stages. Thin arows represent movement between different incarceration stages; the thick arrow at the top represents entry into the model as above and the distribution of people by incarceration status at their entry into the OAT program

Receipt of OAT was represented through five stages: (i) first month on OAT, (ii) short (1–9 months) and (iii) longer (9+ months) duration on OAT, (iv) first month following OAT discontinuation and (v) rest of time following OAT discontinuation (Figure 1 and Supporting information, S1.2, ‘OAT cessation’). This disaggregation aimed to capture the impact of OAT on reducing overdose and other cause mortality, as well as the increased risk of overdose death in the first month of OAT initiation (compared to rest of time on OAT) or discontinuation (compared to rest of time off OAT) [5, 26, 27]. Additionally, as implemented by others [28], it incorporated heterogeneity in OAT durations, representing two main patterns of OAT engagement (rapid turnover and stable treatment), leading to more and less frequent exposure to the risks associated with OAT initiation and discontinuation [29].

Given the high risk of overdose upon release from prison during the first month and up to the first year post‐release, and differential access to OAT in prison, the population was stratified into (i) never or not incarcerated in the past year, (ii) currently incarcerated, (iii) released from prison within the past month and (iv) released from prison 1–12 months ago. The increased risk of overdose and other cause mortality was high during the first month post‐release, and a lower residual increased risk of fatal overdose (but not other cause mortality) was maintained during the remaining 11 months. Mortality from overdose and other causes during incarceration was lower than found outside prison (see Table 1 in the ‘Model parameterization’ section).

**TABLE 1:** Key parameter priors, sampling distributions, sources and posteriors to model the cohort of people receiving opioid agonist treatment in New South Wales, Australia

Variables	Priors	Distribution	Reference	Posteriors
Mortality				
Mortality rate from overdose per year in 2001^b^	Min = 0.001–max = 0.05	Uniform	Wide uncertainty based on 21, 22, 23, 24, 25, 26, 27, 28, 29, 30, 31	0.003 (95% CI = 0.002–0.005)
Mortality rate from other causes among PWUO per year in 2001^b^	Min = 0.001–max = 0.05	Uniform		0.009 (95% CI = 0.007–0.012)
Incarceration patterns and effect on mortality				
RR OD mortality among those released in past month compared to never/not incarcerated in the past year	3.70 (95% CI = 2.22–5.18)	Log‐normal^a^	32	4.032 (95% CI = 3.732–4.358)
RR OD mortality in the 2nd to 12th month post‐release compared to never/not incarcerated in the past year	1.70 (95% CI = 1.02–2.38)	Log‐normal^a^	32	1.161 (95% CI = 1.045–1.341)
RR other cause mortality among those released in the past month compared to never/not incarcerated in the past year	1.40 (95% CI = 0.84–1.96)	Log‐normal^a^	32	1.657 (95% CI = 1.558–1.814)
OAT engagement and effect on mortality				
RR overdose mortality on OAT in the community (compared to off OAT in the community)	0.22 (95% CI = 0.13–0.35)	Log‐normal^a^	5	0.174 (95% CI = 0.147–0.203)
RR other cause mortality on OAT in the community (compared to off OAT in the community)	0.57(95% CI = 0.34–0.80)	Log‐normal^a^	5	0.374 (95% CI = 0.343–0.415)
RR overdose and other cause mortality in the first month on OAT (compared to rest of time on OAT)	1.97 (95% CI = 0.93–4.00)	Log‐normal^a^	5	2.294 (95% CI = 1.926–2.71)
RR overdose and other cause mortality in the first month off OAT (compared to rest of time off OAT)	2.38 (95% CI = 1.53–3.75)	Log‐normal^a^	5	1.782 (95% CI = 1.459–2.394)
RR overdose mortality on OAT in prison (compared to off OAT in prison)	0.12 (95% CI = 0.06–0.26)	Log‐normal^a^	32, 33, 34	0.173 (95% CI = 0.15–0.211)
RR other cause mortality on OAT in prison (compared to off OAT in prison)	0.32 (95% CI = 0.15–0.63)	Log‐normal^a^	32, 33, 34	0.466 (95% CI = 0.401–0.565)
RR of reincarceration on OAT (compared to off OAT)	0.80 (95% CI = 0.71–0.90)	Log‐normal^a^	35	0.818 (95% CI = 0.792–0.84)

OAT = opioid agonist treatment; PWUO = people who use opioids; RR = relative risk; OD = overdose; CI =confidence interval.

‘Priors’ and ‘posteriors’ correspond to the parameter distributions pre‐ and post‐model calibration, respectively.

^**a**^ Priors with lognormal distribution were truncated to the lower and upper prior boundaries.

^b^ Linear increase of mortality rate from overdose and other causes was assumed and full details provided in the Supporting information.

Individuals newly entered the model into the ‘first month on OAT’ compartment and were distributed into the four incarceration status groups described above. Once they had discontinued OAT they could be re‐enrolled multiple times, either in the community or in prison. Individuals either left the model through fatal overdose, other cause mortality or progressed to a ‘no opioid use’ compartment (through ceasing both OAT and illicit opioid use), in which they were only exposed to other cause mortality. Individuals could relapse by transitioning back into opioid use off OAT, and could be re‐enrolled onto OAT. Both overdose and other cause mortality were assumed to increase linearly to reproduce observed increases in these rates over time (see Supporting information, Table S1 and ‘Sensitivity and uncertainty analyses’ section).

### Model parameterization

The model was primarily parameterized using 2001–18 data from the NSW OAT program, obtained through the Electronic Recording and Reporting of Controlled Drugs (ERRCD) as well as from the published literature, including key studies by Sordo *et al*., Degenhardt *et al*. and Larney *et al*. on mortality among people who use opioids [30], OAT effect on mortality through different periods of risk [5, 31] and OAT effect in reducing incarceration [32, 33, 34]. Incarceration data represented all incarceration episodes, including on remand (i.e. pre‐sentence) and custodial sentences of any duration. The majority of parameters were sampled from prior uncertainty distributions (Table 1 for key parameters and Supporting information, Table S1 for full list), with the exception of initial conditions and yearly cohort entries, which were directly based on observed data (Supporting information, Table S2). Full details are provided in the Supporting information.

### Model calibration

We used an approximate Bayesian computation sequential Monte Carlo approach [35] for calibration to incorporate parameter uncertainty. The model was calibrated by minimizing the sum of least squares to the following data normalized to the same scale (Supporting information, Table S3): total number of people in the cohort on 1 January of each year (2001–18), annual number of deaths (2001–17), proportion of overdose‐related deaths per year (2001–16), proportion of overdose‐related deaths while on OAT (2001–16), number of individuals on OAT in prison and out of prison on 1 January each year (2001–18), proportion currently incarcerated on 1 January of each year (2001–18) and proportion ever incarcerated in the past year (excluding those released under 1 month ago) on 1 January of each year (2001–18). One thousand model parameter sets were randomly sampled from parameter prior uncertainty distributions (Supporting information, Table S1) and perturbed over multiple generations, improving model fits at each generation, until successive iterations did not sufficiently improve the model fits. This generated 1000 calibrated model fits, which were used to produce model projections and associated credible intervals (corresponding to the 2.5 and 97.5 percentiles and noted ‘95% CrI’ hereafter).

### Analyses

We implemented a series of analyses to estimate the impact of the OAT program and of specific periods of heightened risk (i.e. OAT initiation, discontinuation and prison release) on opioid overdose and other cause mortality from 2001 to 2020 within the cohort of individuals who ever received OAT during this time‐period (Table 2).

**TABLE 2:** Summary of modeling analyses and detail on implementation and outcomes

Scenario	Purpose	Implementation	Outcome
No OAT program	Estimate the total impact of the NSW OAT program on reducing overdose and other cause deaths	Relative risks of OAT in reducing overdose and other cause mortality set to 1	Proportion of overdose and other cause deaths averted by the NSW OAT program between 2001 and 2020
		Relative risks of OAT in increasing mortality during the first month on and off OAT set to 1	
		Relative risk of OAT in decreasing incarceration set to 1	
No OAT program in prison	Estimate the impact of the prison OAT program on reducing overdose and other cause deaths	OAT enrolment rate in prison set to 0	Proportion of overdose and other cause deaths averted by the prison OAT program between 2001 and 2020
		Progression rate from ‘on OAT’ to ‘off OAT’ in prison set to 100/year	
		All new cohort members entering through prison assigned to the ‘off OAT’ compartment	
No increased mortality risk in the first month post‐prison release	Estimate the contribution of the first month post‐incarceration release period to overdose and other cause mortality	Relative risk of overdose and other cause mortality during the 1st month post‐incarceration release set to 1	Population‐attributable fraction of the first month post‐incarceration release associated risks to overdose and other cause mortality between 2001 and 2020
No increased mortality risk in the first month on OAT (i.e. induction)	Estimate the contribution of the OAT induction period to overdose and other cause mortality	Relative risk of overdose and other cause mortality during the 1st month on OAT set to 1	Population‐attributable fraction of OAT induction associated risks to overdose and other cause mortality between 2001 and 2020
No increased mortality risk in the first off OAT (i.e. discontinuation)	Estimate the contribution of the OAT discontinuation period to overdose and other cause mortality	Relative risk of overdose and other cause mortality during the 1st month off OAT set to 1	Population‐attributable fraction of OAT discontinuation associated risks to overdose and other cause mortality between 2001 and 2020

OAT = opioid agonist treatment.

To estimate the impact of the community and prison OAT program, we compared mortality (overdose and all cause) for the calibrated scenario with OAT from 2001 to 2020 (‘baseline scenario’) to a scenario assuming no OAT effect on mortality, implemented through setting the OAT overdose and other cause mortality risks in the first month on and off and during treatment equal to 1, and the relative risk of incarceration on OAT to 1. The latter was implemented to account for the fact that through reducing incarceration risk, OAT also reduces exposure to heightened mortality risk during the post‐release period.

We then assessed the specific contribution of the prison OAT program to the overall OAT program impact by comparing the baseline scenario to a scenario assuming no OAT program in prison, implemented through moving people in prison OAT to off OAT, interrupting OAT upon incarceration and stopping OAT enrollment while in prison. Importantly, through removing prison OAT provision, more people are exposed to the increased mortality risk in the first year following prison release. To estimate the number of deaths averted/100 person‐years on OAT in the first year post‐release, achieved through prison OAT provision, we divided the number of additional deaths during this first year post‐release by the missed number of OAT person‐years during this period in this scenario versus baseline (and also estimated it specifically for the first month post‐release).

We additionally estimated the contribution (i.e. population attributable fraction) of three high‐risk periods (first month post‐incarceration release and first month of OAT initiation and discontinuation) to overdose and other cause mortality from 2001 to 2020. We assessed the contribution of first month post‐incarceration release by comparing the baseline scenario to a scenario where the relative risks of death associated with the first month post‐incarceration release were set to 1. We assessed the contribution of increased risk upon the first month of OAT initiation and discontinuation by comparing the baseline scenario to a scenario where the relative risk of increased overdose and other cause mortality during the first month of OAT initiation or discontinuation, respectively, were set to 1.

### Sensitivity and uncertainty analyses

We implemented two sensitivity analyses to investigate the impact of assumptions on predicted proportion of deaths averted through the OAT program. First, both linear and exponential functions fitted the observed overdose data trends. In our main analyses we conservatively assumed a linearly increasing trend, therefore we also implemented a sensitivity analysis assuming an exponentially increasing overdose mortality rate. Secondly, we performed an analysis assuming no relapse to opioid use after prolonged cessation. We implemented an analysis of covariance to identify parameters that contributed most to uncertainty in projections of deaths averted. All the data used for the modeling study are provided in the Supporting information.

## RESULTS

### Cohort data from 2001 to 2018

A total of 49 359 people received OAT at least once between 2001 and 2018 in NSW, with an average of 16 886 (range across years = 13 889–20 048) receiving OAT at any one time, resulting in an average OAT coverage of 52% (range across years = 47–66%) among this cohort and 312 756 observed person‐years on OAT. On average, individuals went through 3.7 OAT episodes, for a mean duration of 1.74 years [95% confidence interval (CI) = 1.68–1.80] per episode and ranging from 1 day to 17.4 years. From 2001 to 2018, at any one time a mean 7.3% (range across years = 5.4–9.2%) of individuals in the cohort were in prison and 23.1% (range across years = 14.0–31.9%) were incarcerated during the past year. From 2001 to 2016 there were a total of 5045 deaths, 1508 of which were overdose deaths (0.9/100 person‐years and 0.6/100 person‐years, respectively) [30].

### Model comparison to data

**FIGURE 2:**
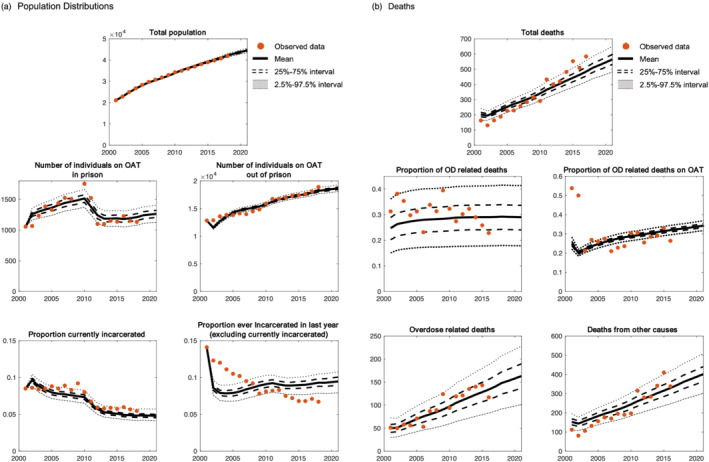
Model calibration outputs and data among the cohort of people receiving opioid agonist treatment (OAT) in New South Wales, Australia. (a) Population distributions; (b) deaths

Our model successfully reproduced the cohort data, suggesting that it captured the primary mechanisms underlying these observations. From 2001 to 2016 the model estimated 4741 (95% CrI = 3913–5572) total deaths and 1304 (95% CrI = 622–1996) overdose deaths. As shown in Figure 2a, the 95% CrIs of projections included most data points for six demographic outcomes, with the exception of the proportion incarcerated in the past year, where estimates were close to data values, but included fewer data points. The model closely fitted the number of people receiving OAT through time, estimating 305 124 (95% CrI = 297 694–313 141] person‐years on OAT from 2001 to 2018. Mean model estimates of number of OAT episodes were 3.7/person for a mean duration of 1.67 years. The model closely reproduced mortality data (Figure 2b), including by cause and OAT status. Model validation against mortality data by incarceration status not used in the calibration process are shown in Supporting information, Figure S2. The model reproduced these data well, except for an increase in overdose upon release from prison from 2011 onwards (see Discussion for potential reasons).

### Impact of OAT program on overdose and other cause mortality

**FIGURE 3:**
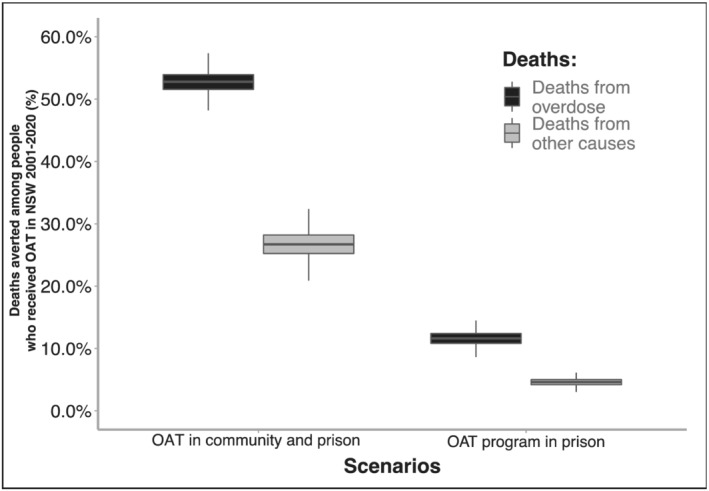
Percentage of overdose and other cause deaths averted among the cohort of people who received opioid agonist treatment (OAT) in NSW between 2001 and 2020 through the full OAT program and the OAT program in prison. See also Supporting information, Figure S4 for relative change in deaths through the full OAT program and the OAT program in prison compared to no OAT provision from 2001 to 2020

The baseline model scenario estimated that there were 2020 (95% CrI = 971–3089) overdose deaths between 2001 and 2020 among people accessing OAT in NSW, which could have increased to 4274 (95% CrI = 2078–6538) overdose deaths with no OAT program, based on counterfactual model projections (Table 3). OAT provision therefore averted an estimated 2254 (95% CrI = 1098–3478) overdose‐related deaths between 2001 and 2020, a 52.8% (95% CrI = 49.4–56.9) reduction (Figure 3). Additional benefits were observed on other cause mortality, with an estimated 5268 (95% CrI = 3694–6693) other cause deaths with OAT provision, increasing to 7181 (95% CrI = 5021–9039) if OAT had not been provided, suggesting that OAT provision led to a 26.6% (95% CrI = 22.1–30.5) reduction in other cause mortality. In total, OAT averted 4166 (95% CrI = 3190–5193) deaths in NSW from 2001 to 2020, corresponding to 33 063 (95% CrI = 27 060–40 371) life years gained (LYG). During 2001–20 we estimated 340 016 person‐years on OAT, corresponding to 1.2 deaths averted and 9.7 LYG per 100 person‐years on OAT.

**TABLE 3:** Predicted number of overdose deaths, other cause deaths and total deaths among people receiving opioid agonist treatment (OAT) in NSW, Australia under different scenarios from 2001 to 2020

	Overdose deaths, 2001–20 Mean (95% CrI)	Change in overdose deaths compared to baseline with OAT Mean (95% CrI)	% Overdose deaths averted, 2001–20 Mean (95% CrI)	Deaths from other causes, 2001–20 Mean (95% CrI)	Change in other cause deaths compared to baseline with OAT Mean (95% CrI)	% Other cause deaths averted, 2001–20 Mean (95% CrI)	Total deaths, 2001–20 Mean (95%)	Change in total deaths compared to baseline with OAT Mean (95% CrI)	% Total deaths averted, 2001–20 Mean (95% CrI)
Baseline (with OAT)	2020 (95% CrI = 971–3089)	–		5268 (95% CrI = 3694–6693)	–		7288 (95% CrI = 6032–8587)	–	
No OAT (prison or community)	4274 (95% CrI = 2078–6538)	2254 (95% CrI = 1098–3478)	52.8 (95% CrI = 49.4–56.9)	7181 (95% CrI = 5021–9039)	1912 (95% CrI = 1295–2493)	26.6 (95% CrI = 22.1–30.5)	11 454 (95% CrI = 9504–13 414)	4166 (95% CrI = 3190–5193)	36.3 (95% CrI = 31.3–41.9)
No prison OAT program	2285 (95% CrI = 1090–3509)	265 (95% CrI = 131–426)	11.6 (95% CrI = 9.4–13.8)	5523 (95% CrI = 3881–6994)	254 (95% CrI = 161–346)	4.6 (95% CrI = 3.5–5.7)	7807 (95% CrI = 6471–9187)	519 (95% CrI = 384–682)	6.7 (95% CrI = 5.1–8.8)
No increased risk first month post‐release	1949 (95% CrI = 940–2979)	−71 (95% CrI = –117 to –34)	3.5 (95% CrI = 2.7–4.6)	5252 (95% CrI = 3681–6671)	−17 (95% CrI = –24 to –10)	0.32 (95% CrI = 0.2–0.4)	7200 (95% CrI = 5952–8497)	−88 (95% CrI = –132 to –53)	1.2 (95% CrI = 0.7–1.8)
No increased risk during first month on OAT	1978 (95% CrI = 917–2885)	−42 (95% CrI –95 to –13)	2.1 (95% CrI = 0.8–4.0)	5209 (95% CrI = 3612–6601)	−59 (95% CrI = –118 to –24)	1.1 (95% CrI = 0.4–2.0)	7187 (95% CrI = 6006–8388)	−101 (95% CrI = –188 to –75)	1.4 (95% CrI = 0.8–2.0)
No increased risk during first month off OAT	1936 (95% CrI = 922–2668)	−84 (95% CrI = –153 to –38)	4.2 (95% CrI = 2.3–7.3)	5121 (95% CrI = 3605–6564)	−146 (95% CrI = –252 to –67)	2.8 (95% CrI = 1.1–4.2)	7058 (95% CrI = 5862–8007)	−230 (95% CrI = –412 to –174)	3.0 (95% CrI = 2.1–5.3)

OAT = opioid agonist treatment; CrI = credible interval.

### Impact of prison OAT program

A scenario assuming no OAT provision in prison, causing both lower OAT enrolment and treatment interruptions, resulted in 265 (95% CrI = 131–426) and 254 (95% CrI = 161–346) more overdose and other cause deaths, respectively, from 2001 to 2020 (Table 3). This equates to 11.6% (95% CrI = 9.4–13.8) and 4.6% (95% CrI = 3.5–5.7) of overdose and other cause deaths, respectively, averted through prison OAT. Overall, the prison OAT program prevented 12.4% (95% CrI = 11.5–13.5) of all the deaths averted by the OAT program in NSW. Without prison OAT, we estimate 47 007 fewer person‐years on OAT among the cohort. Importantly, there were 11 940 fewer OAT person‐years during the first year post‐incarceration release, translating into 1.8 deaths averted per 100 person‐years on OAT during this high‐risk period. Focusing on the first month post‐release only, this corresponds to 3.4 deaths averted per 100 person‐years on OAT.

### Population‐attributable fraction of first month post‐incarceration release

**FIGURE 4:**
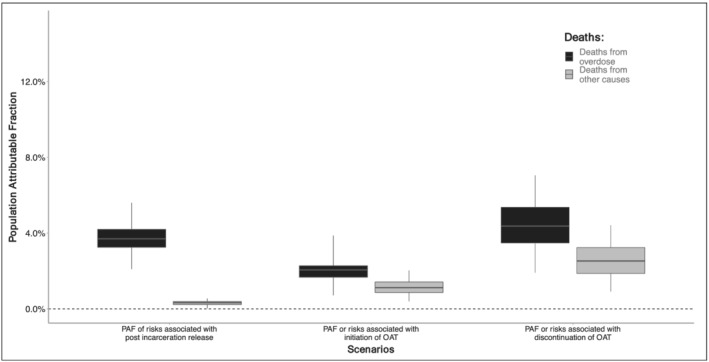
Population‐attributable fraction (PAF) of first month post‐incarceration release period and opioid agonist treatment (OAT) induction/cessation on overdose and other cause mortality among the cohort of people who received OAT in NSW between 2001 and 2020. See also Supporting information, Figure S4 for relative change in death due to incarceration and OAT initiation/discontinuation on overdose and other cause mortality in NSW from 2001 to 2020

We estimated the elevated risk in the first month post‐release contributed to 71 (95% CrI = 34–117) overdose deaths or 3.5% (95% CrI = 2.7–4.6) of overdose‐related mortality and 17 (95% CrI = 10–24) other cause deaths (Table 3 and Figure 4).

### Population‐attributable fraction of OAT initiation and discontinuation

We estimated the increased risk in the first month following OAT initiation and cessation contributed to 42 (95% CrI = 13–95) and 84 (95% CrI = 38–153) overdose deaths and to 59 (95% CrI = 24–118) and 146 (95% CrI = 67–252) deaths from other causes, respectively. Interventions addressing the excess risk during OAT initiation and cessation could have prevented up to 1.4% (95% CrI = 0.8–2.0) and 3.0% (95% CrI = 2.1–5.3) of all deaths, respectively (Table 3 and Figure 4).

### Sensitivity analyses

Assuming an exponentially increasing overdose mortality rate resulted in a marginally higher impact of the OAT program on overdose deaths (55.6% relative reduction compared to 52.8% at baseline), but a lower impact on other cause deaths (22.4% relative reduction compared to 26.6% at baseline). Assuming no relapse resulted in a marginally lower impact of the OAT program (50.7% relative reduction in overdose deaths, 21.1% relative reduction in other cause deaths 2001–20); see Supporting information, Table S6.

Analyses of variance showed that parameters associated with greater uncertainty in our model predictions of total deaths averted during 2001 to 2020 were those which determined the community effect of OAT on mortality: (1) the effect of OAT on overdose mortality in the community (56.3% of variance explained), (2) the effect of OAT on other cause mortality in the community (20.4% of variance) and (3) increased mortality in the first month on OAT (9.2% of variance) (Supporting information, Table S7).

## DISCUSSION

In this study, we presented the first dynamic model assessing the population‐impact of a large‐scale OAT program in NSW on mortality over 20 years. Our model reproduced time trends in OAT, incarceration and mortality patterns among a state‐wide cohort of people with OUD, using linked data across sectors. Overall, the OAT program in NSW averted more than 4000 deaths between 2001 and 2020 among 49 359 people who received treatment at least once during this period through preventing 53% of overdose deaths and 27% of other cause deaths, corresponding to a 36% reduction in overall mortality. More than 33 000 life years were gained, with nearly 10 life years gained per 100 person‐years on OAT. State‐wide, 2582 opioid‐related overdose deaths occurred from 2001 to 2016 [36], and 1317 of these among this cohort. This suggests that the OAT program averted more than a third of all opioid overdose deaths in NSW and more could probably have been prevented if more people with OUD had received OAT. Our model also showed that, through preventing treatment interruption, OAT provision in prison averted approximately 12% of all deaths averted by the OAT program.

### Comparison with other studies

Our study updates an earlier statistical analysis that estimated a 29% reduction in mortality among people on OAT from 1985 to 2006 [31] in NSW by applying the observed crude mortality rate among those off OAT to the total number of person‐years in the cohort. Instead, our dynamic modeling simulated system dynamics of OAT engagement, incarceration, opioid use cessation and relapse. In particular, different patterns of OAT engagement affect mortality risk, with frequent OAT interruptions and re‐initiations leading to higher mortality compared to long/stable treatment duration [26]. Effective overdose prevention interventions during the induction and discontinuation periods could have prevented up to 2.1% and 4.2% of overdose deaths, respectively. Peer, social worker [37] and telehealth interventions [38, 39] to support patients during the induction period, as well as distress tolerance interventions [40] and novel regimens such as microdosing [41, 42, 43] to prevent precipitated withdrawal are being investigated and could potentially reduce overdose risk during this period. For some patients, residential treatment [44] during the first week(s) of treatment will be warranted to prevent early dropout associated with withdrawal symptoms. Preventing overdose during the first month following OAT discontinuation is more challenging, given that discontinuation is often related to relapse as a result of difficult and chaotic life events. Regular overdose education and naloxone distribution (OEND) among patients, but also their family members, partners and friends delivered as part of OAT programs, could help to prevent fatal overdoses during relapse. Contact with emergency services, law enforcement and penitentiary institutions is high during OAT discontinuation [45] and is predictive of non‐fatal and fatal overdose [46], highlighting the importance of strong overdose prevention programs in these settings.

Generally, our findings are consistent with other dynamic model projections predicting the potential impact of scaling‐up OAT (particularly if long duration) on overdose mortality among people who inject drugs (PWID) in Russia, Mexico, United States and Iran [9, 47, 48, 49], and also support previous ecological analyses which found associations between expansion of OAT programs and reduced mortality [15, 16, 17, 19]. A recent dynamic modeling study using US Veteran Health Administration data found that treatment duration had to be more than 6 months to ensure a net mortality benefit of OAT across nearly all model projections, with longer treatment durations leading to increased impact [50]. In contrast, estimates in the United Kingdom suggested that an average duration of OAT of more than 1 year may be required to reduce overdose deaths in the population, and showed substantially poorer retention in England compared to NSW (1.74 years in NSW versus 1 and 0.47 years for methadone and buprenorphine, respectively, in England [19, 26]). OAT discontinuation has been associated with more severe OUD (i.e. higher frequency of opioid use), polysubstance use and younger age, as well as precarious living conditions, including homelessness, incarceration and lack of social income assistance [51, 52, 53]. Both type of OAT medication and dose [51, 54] must be well adapted to patients’ OUD severity. While buprenorphine and buprenorphine–naloxone prescription have been associated with higher rates of discontinuation versus methadone prescription [52], others have reported contrasting findings [55] and large‐scale studies are being implemented to guide clinical practice [24, 56, 57]. Interventions to improve medication regimen choice through better assessment of symptoms have been proposed [58], and strategies to increase retention through counselling, therapeutic drug monitoring [59] and contingency management are being tested [59, 60] and have shown promise [61]. Providing stable housing and employment opportunities support the recovery process beyond OAT retention through promoting personal satisfaction and social integration. In addition, addressing fear of health‐system or medication dependence as well as stigma [62, 63] towards long‐term OAT, especially methadone, is also key to improving OAT retention.

The mortality risk among people with OUD is heterogeneous and higher upon release from incarceration. Our estimates of the proportion of overdose deaths during the first month following prison are lower than other estimates—i.e. < 4% in NSW compared to more than 7% in Scotland [64], probably due to high OAT coverage in NSW prisons. Our estimates of prison OAT impact on fatal overdose in NSW are double that found by Macmadu *et al*. [65], which estimated that access to medications for OUD at release from prisons and jails in Rhode Island, USA could prevent 5.8% of opioid overdose deaths during 2017–24. Differences can be partially attributed to their lower assumption of OAT efficacy on overdose (relative risk of 0.22 in NSW versus 0.40 in RI) and differences in retention on OAT (1.74 years in NSW versus 1 year in RI). We note that OAT enrolment in NSW prisons decreased during 2016–18 and the OAT regimen in prison consisted of providing treatment 2 weeks pre‐release (versus through the entire incarceration period), probably leading to higher dropout rates post‐release and potentially explaining the higher number of overdose deaths observed during the first month post‐release (Supporting information, Table S2C and Figure S2). The increase in post‐release fatal overdoses could also be due to changes in the post‐release environment, such as higher prevalence of unstable housing [66, 67], leading to engagement in higher‐risk behaviors or changes in drug markets linked to more dangerous polydrug use. In both cases, this points towards the need for more radical interventions to address the excess risk during post‐incarceration release [68, 69]. Fortunately, depot buprenorphine is currently been scaled‐up in NSW prisons [70] and might revert this trend. Indeed, OAT provision in prison has been shown to be highly effective at reducing fatal overdose post‐release [6] and should be made available in correctional settings where it currently is not [71]. In addition, providing support during the month preceding release through engagement with recovery mentors and connection with community OAT services [72] has proved successful in facilitating treatment continuity, and findings from the impact evaluation of the ‘Connections’ program in NSW prisons on reducing mortality, recidivism and improving parenting outcomes will shed further light on these interventions [73]. OEND in correctional settings has been shown to be acceptable and feasible and to increase trainees’ self‐efficacy to respond to an overdose [74]. However, demonstrating effectiveness at reducing fatal overdose among incarcerated individuals upon release has proved challenging, given that the majority of naloxone doses distributed are administered to someone else other than the trainee. Other studies have provided OEND to visitors in prison and shown that the naloxone kits distributed reach communities of both high overdose and high incarceration rates [74].

### Limitations

Our study has several limitations. First, our analysis is restricted to the cohort of people who received OAT. With > 40% and > 60% of all PWID in NSW reporting current and life‐time OAT use [21, 22, 23], the cohort probably represents a high proportion of all people with OUD, but current and historical estimates of the prevalence of opioid use and injection are not yet robust enough to accurately estimate the proportion exposed to OAT and the proportion of deaths averted among the total population with OUD. As such, further analyses are needed to assess impact of OAT scale‐up to those not reached by the program. Secondly, there is uncertainty regarding the model parameters which we incorporated into our analysis, but which generated associated uncertainty in the outcomes. For example, OAT treatment end‐dates in the cohort are probably less reliable than treatment start‐dates, as reporting relies upon treatment center reports. Nonetheless, we successfully reproduced OAT coverage over time as well as deaths on and off OAT, suggesting that our model captured OAT engagement patterns in this cohort. Data on deaths outside NSW are not available from 2010 to 2018, and we therefore applied a 10% increase in observed deaths based on both data from the cohort previous to 2010 and on Census data, potentially leading to over‐ or under‐estimation of the OAT program's impact. Thirdly, we did not account for differences in OUD severity or mode of administration (i.e. injected use versus not). Unfortunately this information is not available from the cohort, but future research should evaluate how differences in access to OAT by OUD severity affect overall program impact. Similarly, we do not explicitly simulate differences by OAT modality (such as methadone versus buprenorphine) or dosage. More detailed analyses examining the relative benefits of different OAT regimens would inform future optimization of services [75]. Fourthly, our study is limited to estimating the impact of the OAT program on deaths, and does not incorporate the reduced risk of HIV or hepatitis C virus while on OAT, nor does it consider reductions in morbidity and increased wellbeing associated with OAT. Beyond individual‐level benefits of OAT, we do not incorporate societal impacts through increases in productivity, crime reduction and improvements in the wellbeing of family and friends. Finally, our model is informed by data from NSW and may not be entirely generalizable to other settings, which may have different patterns of OAT delivery and client characteristics (including incarceration rates), but it shows the potential of OAT to prevent deaths in the population if scaled‐up at high coverage and with good retention in the community and prisons. Setting specific evaluations of OAT programs’ impact on premature mortality at population level are key to inform their delivery and improve their effectiveness.

## CONCLUSION

The OAT program in NSW has averted 53% of overdose deaths among clients receiving OAT between 2001 and 2020 and a third of all overdose deaths in NSW. OAT coverage and retention is poor among people using opioids in many countries and needs to be improved to tackle the public health crisis of increasing drug‐related deaths world‐wide.

## DECLARATION OF INTERESTS

A.B. has no conflicts of interest to declare. N.M. has received unrestricted reserach grants from Merck and Gilead unrelated to this work. L.D. and M.F. have received investigator‐initiated untied educational grants for studies of opioid medications in Australia from Indivior, Mundipharma and Seqirus. S.L. has received untied educational grant funding from Indivior unrelated to this work.

## ETHICS AND APPROVALS

This study is approved by the NSW Population and Health Services Research Ethics Committee (2018/HRE0205) and Aboriginal Health and Medical Research Council Research Ethics Committee (1400/18).https://ndarc.med.unsw.edu.au/project/opioid-agonist-treatment-and-safety-oats-study.

## AUTHOR CONTRIBUTIONS

**Antoine Chaillon:** Conceptualization; data curation; formal analysis; investigation; visualization. **Chrianna Bharat:** Data curation; formal analysis; investigation. **Jack Stone:** Conceptualization; formal analysis; investigation; methodology. **Nicola Jones:** Data curation; formal analysis. **Louisa Degenhardt:** Conceptualization; funding acquisition; investigation; resources; supervision. **Sarah Larney:** Funding acquisition; investigation; resources; supervision. **Michael Farrell:** Funding acquisition; investigation; resources; supervision. **Peter Vickerman:** Conceptualization; investigation; methodology; supervision. **Matthew Hickman:** Conceptualization; investigation; supervision. **Natasha Martin:** Conceptualization; formal analysis; investigation; methodology; supervision. **Annick Borquez:** Conceptualization; formal analysis; investigation; methodology; supervision.

## Supporting information


**Table S1.** Parameter priors, sampling distributions, sources and posteriors to model the cohort of people receiving opioid agonist treatment (OAT) in New South Wales. PWUO: people who use opioids. RR: relative risk. OD: overdose.
**Table S2.** Initial population distribution in 2001 and yearly new entries. A. Initial cohort population distribution by incarceration status. B. Initial population distribution by opioid agonist treatment (OAT) status. C. Yearly new opioid agonist treatment (OAT) cohort entries by incarceration status
**Table S3.** Calibration data. OAT: opioid agonist treatment, NA: not available
**Table S4.** Overdose death rate per year from 2000 to 2016 by years since first entry into opioid agonist treatment (OAT) from the cohort data.
**Table S5.** Calibrated opioid agonist treatment (OAT) transition rates weighted by the proportion on buprenorphine and methadone, including +/−20% uncertainty to obtain a prior distribution.
**Table S6.** Sensitivity analyses to evaluate the impact of OAT programs assuming (A) exponentially increasing overdose mortality rate and (B) no relapse. A. Mortality rate from overdose per year $\mu_{1}=\mu_{1}\times \exp\left(0.11\left(\tau -\tau_{\text{start}}\right)\right)$ with $\tau_{\text{start}}=2001$
**Table S7.** Ancova analysis. OAT: opioid agonist treatment, RR: relative risk
**Figure S1.** Comparison of the calibrated exponential function (black line) to data (red line) on the retention of individuals on opioid agonist treatment for buprenorphine (A) and methadone recipients (B).
**Figure S2.** Model validation against data not used for the calibration. OD: overdose
**Figure S3.** Prior and posterior parameter distributions. Red area denotes the prior distribution and vertical dotted lines show the lower and upper limits of prior values. Posterior distributions are depicted by gray bars. Priors with lognormal distribution were truncated to the lower and upper prior boundaries. OAT: opioid agonist treatment.
**Figure S4.** Relative change of overdose and other cause deaths averted among the OAT cohort in NSW (A) through the full OAT program and the OAT program in prison compared to no OAT provision and (B) due to post‐incarceration release, no increased risk during first month on OAT and no increased risk during first month off OAT from 2001 to 2020. OAT: opioid agonist treatment
