# Supplementary material for: Modeling the population‐level impact of opioid agonist treatment on mortality among people accessing treatment between 2001 and 2020 in New South Wales, Australia
Source: Addiction. 2021 Dec 4;117(5):1338–52. doi: 10.1111/add.15736 (PMC9299987; doi:10.1111/add.15736)
Supplement: Supplementary file 1 — Table S1. Parameter priors, sampling distributions, sources and posteriors to model the cohort of people receiving opioid agonist treatment (OAT) in New South Wales. PWUO: people who use opioids. RR: relative risk. OD: overdose. Table S2. Initial population distribution in 2001 and yearly new entries. A. Initial cohort population distribution by incarceration status. B. Initial population distribution by opioid agonist treatment (OAT) status. C. Yearly new opioid agonist treatment (OAT) cohort entries by incarceration status Table S3. Calibration data. OAT: opioid agonist treatment, NA: not available Table S4. Overdose death rate per year from 2000 to 2016 by years since first entry into opioid agonist treatment (OAT) from the cohort data. Table S5. Calibrated opioid agonist treatment (OAT) transition rates weighted by the proportion on buprenorphine and methadone, including +/−20% uncertainty to obtain a prior distribution. Table S6. Sensitivity analyses to evaluate the impact of OAT programs assuming (A) exponentially increasing overdose mortality rate and (B) no relapse. A. Mortality rate from overdose per year μ1=μ1×exp0.11τ−τstart with τstart=2001 Table S7. Ancova analysis. OAT: opioid agonist treatment, RR: relative risk Figure S1. Comparison of the calibrated exponential function (black line) to data (red line) on the retention of individuals on opioid agonist treatment for buprenorphine (A) and methadone recipients (B). Figure S2. Model validation against data not used for the calibration. OD: overdose Figure S3. Prior and posterior parameter distributions. Red area denotes the prior distribution and vertical dotted lines show the lower and upper limits of prior values. Posterior distributions are depicted by gray bars. Priors with lognormal distribution were truncated to the lower and upper prior boundaries. OAT: opioid agonist treatment. Figure S4. Relative change of overdose and other cause deaths averted among the OAT cohort in NSW (A) through the full O [file ADD-117-1338-s001.docx]

**TITLE: Modeling the population level impact of opioid agonist treatment on mortality among people accessing treatment between 2001-2020 in New South Wales, Australia**

**AUTHORS:** Chaillon A^1^, Bharat C^2^, Stone J^3^, Jones N^2^, Degenhardt L^2^, Larney S^2,4^, Farrell M^2^, Vickerman P^3^, Hickman M^3^, Martin NK^1,3^*, Bórquez A^1,2^*

*equally contributing senior authors

**AFFILIATIONS:**

1. Division of Infectious Diseases and Global Public Health, University of California, San Diego, US
2. National Drug and Alcohol Research Centre, University of New South Wales, Australia
3. Population Health Sciences, University of Bristol, United Kingdom
4. Centre de Recherche du Centre Hospitalier de l'Université de Montréal (CRCHUM) and Department of Family Medicine and Emergency Medicine, Université de Montréal

**SUPPLEMENTARY MATERIALS**

1. **Model parameterization**

The Electronic Recording and Reporting of Controlled Drugs (ERRCD) data^1^ has been linked to mortality data from the Registry of Births and Deaths (RBDM), and to incarceration data from the NSW Bureau of Crime Statistics and Research (BOCSAR), through project OATS (Opioid Agonist Treatment Safety, described elsewhere^2^). All model parameters are provided in **Table S1**, while initial conditions and distributions at entry to the OAT program (and model) which were directly taken from the cohort data are shown in **Table S2**.

*1.1 Demographic parameters*

Initial conditions and yearly entries: The initial size and distribution of the population across incarceration and OAT status compartments was determined based on data from OAT recipients on January 1^st^ 2001 (**Table S2-A and B**). Yearly new entries in the cohort in 2001-2018 were based on the total number of people newly entering the OAT program each calendar year^2^. Individuals entered into the first month on OAT and were distributed by incarceration status based on yearly data (**Table S2-C**).

Overdose and other cause mortality: Individuals currently using opioids were exposed to both overdose and other cause mortality, while those not currently using opioids were only exposed to other cause mortality (at a different rate compared to those currently using). Both overdose and other cause mortality were assumed to increase linearly to reflect observed increases in these rates over time (see footnote **Table S1** for equations). The latter can be explained through aging of our cohort, while the former suggests the risk of fatal overdose has increased over time among this cohort. In sensitivity analyses (**Table S6**), we explored the impact of assuming an exponential versus a linear increase in overdose death rates. Given mortality is lower in prison than in the community, we applied a relative risk of overdose and other cause mortality in prison using broad priors based on the cohort data ^3,4^.

Opioid use cessation and relapse: We incorporated long term opioid use cessation using broad priors around estimates from Calabria et al.^5^. The relatively high rates of overdose mortality among people who entered the cohort over 10 years ago (**Table S4**) suggest a high proportion were still using opioids after many years since their first treatment episode, reflecting either very long durations of opioid use or relapse among this group. We incorporated a low relapse rate in order to acknowledge this eventuality even among people with long term cessation^6-10^.

*1.2 OAT parameters*

OAT cessation: As OAT dropout is higher in the first few months of treatment, we determined OAT attrition from program data as in Mukandavire *et al*^11^ where a split exponential function was fit to proportion retained on OAT over time (for both buprenorphine and methadone recipients), generating an estimation of the average OAT dropout from short and long OAT episodes (**Table S5** and **Figure S1**). The form of the exponential function used was as follows:

$\left. Proportion retained on OAT = \left\{ \begin{aligned} \exp\left( -\kappa t \right), if t<a, \\ \exp\left( -\kappa t*a \right)*\exp\left( -\pi\left( t-a \right) \right), if t\geq a \end{aligned} \right. \right\}$ (1)

This function assumes two attrition rates associated with OAT, with clients leaving OAT at a rate $\kappa$ for the initial period on OAT up to time $a$, and then after that leaving OAT at a rate $\pi$. The exponential curves obtained from the fitting equation to data from the ASCEND study (using Matlab solver to minimise the sum of the squared error) are shown in **Supplementary Figure S1** below.

As shown in **Table S1**, the posterior mean for the transition rate from short to long OAT is 1.49/year, which corresponds to an average duration of 8 months in short OAT before moving to long OAT. Considering that getting to the short OAT compartment requires transitioning from the first month OAT induction period, those moving to the long OAT compartment will have received OAT for 1+8=9 months. They can however drop out at any point between entering this short OAT period after the 1 month induction and transitioning to long OAT after 9 months. The posterior mean for the dropout rate from long OAT is 0.163/year, corresponding to an average duration of to 6.1 years in long OAT. Given reaching long OAT required transitioning from short OAT, those leaving the long OAT compartment will have received OAT for 9 months + 6.1 years.

Therefore those in the induction period stay on OAT for up to 1 month before either dropping out of transitioning to short OAT, those in short OAT receive treatment for at least one month and up to 9 months before either dropping out or transitioning to long OAT and those in long OAT receive treatment for at least 9 months and for 6.9 years on average.

OAT re-enrollment: Individuals off OAT could be recruited back on OAT with setting-specific (community or prison) rates calibrated to the 2001-2018 NSW data on OAT recipients within each setting, as described in the calibration section. Given the observed increase in the community OAT re-enrolment rate from 2001-2018, we applied an increasing linear function to these recruitment rates through time (from 2001-2020).

Relative risk of overdose and other cause mortality on OAT in the community and prison: Prior distributions of the relative risk of overdose-related death when on OAT (compared to off OAT for over one month) was estimated from an international systematic review and meta-analysis (0.21 [95%CI 0.12-0.35])^12^. The risk of other cause mortality on OAT (0.57 [95%CI: 0.34-0.80] compared to off OAT), elevated relative risk of overdose and other cause mortality on OAT in the first month (1.97 [95%CI:0.93-4.00] compared to on OAT after the first month), and elevated risk of overdose and other cause mortality in the first month off OAT (2.38 [95%CI:1.53-3.75] compared to off OAT after the first month) were obtained from the same study^12^. The meta-analysis included data from our cohort, but we used the pooled estimates as these had broader confidence intervals. Given the delivery of OAT is different in prison versus the community (i.e. daily directly observed treatment versus not), we estimated the effect of OAT on reducing overdose mortality (0.12 [95%CI 0.06-0.26]) and other cause mortality (0.32 [95%CI: 0.15-0.63]) in prison based on the cohort data. There were no overdose deaths on OAT in prison over the studies period and therefore we assumed a very low relative risk assuming the mean was equal to the low bound of the relative risk of overdose mortality on OAT obtained from the meta-analysis above (i.e. 0.12) and arbitrarily applying the same variance using the log-normal distribution to calculate confidence interval. To calculate the relative risk of other cause mortality on OAT we used data from the cohort presented in Larney et al^3^ (i.e. RR=(10/15377)/(27/12462)).

*1.3 Incarceration parameters*

(Re)-incarceration rates and exit from prison: Based on prior studies, we assume a lower incarceration rate among those never or not incarcerated in the past year compared to those incarcerated in the past year, which was implemented through applying a relative risk of incarceration for the never or not incarcerated in the past year group with broad priors of 0-1. Approximately 40% of those never incarcerated had never been incarcerated and primary incarceration is much lower than reincarceration, which motivated this assumption. We validated the posterior incarceration rate among this group with data in the validation section. We assumed the re-incarceration rate among those incarcerated in the past month was equal to that among those incarcerated in the past year. In order to reproduce the decline in the number of people incarcerated after 2010, we applied a higher incarceration rate from 2001 up to 2010 and lower from 2010 onwards. The prison exit rate was estimated based on the mean duration of incarceration in the cohort (approx. 10 months)^2^. Incarceration rates among those not using opioids (i.e. opioid use cessation) were assumed to be lower than among those currently using opioids. We based the low bound of this relative risk on the relative incarceration risk among the total population and the high bound on the relative risk of incarceration among people currently using opioids on OAT.

Increased risk of overdose and other cause mortality associated with incarceration: The priors for the relative risk of overdose and other cause mortality among those in prison compared to in the community were calculated directly from the cohort (0.05 [95%CI: 0.02- 0.14) and 0.31 [95%CI: 0.19- 0.47), respectively) as no other data are available^13^. These were calculated from two studies as shown below:

RR overdose mortality in prison versus outside (off OAT):

6/12462 overdose deaths off OAT in prison^3^

133/14222 overdose deaths off OAT in first year post release^4^

RR other cause mortality in prison versus outside (off OAT):

27/12462 other cause deaths off OAT in prison^3^

117/16286 other cause deaths off OAT in first year post release^4^

Alternative estimate: 1528/201073 other cause deaths off OAT among all (including in prison)^14^

We took the average between the two estimates as our point estimate and lowest and highest bound from each. These two estimates were very similar though.

The relative increase in overdose mortality among those released from prison in the past month and past year, compared to those never/not incarcerated in the past year was 3.7 [95%CI: 2.22-5.18] and 1.7 [95%CI: 1.02-2.38], respectively, based on Degenhardt^4^. Similarly, the relative risk of mortality from other causes among those released from prison in the past month was 1.4 [95%CI: 0.84-1.96], with no difference among those released from prison in the past year^4^.

Decreased risk of incarceration while on OAT: the prior relative risk of incarceration/re-incarceration on OAT was 0.8 [95%CI: 0.71–0.90]^15^ compared to those off OAT.

1. **Model Calibration**

The data used to calibrate the model are provided in **Table S3**, and prior and posterior distributions for all parameters are presented in **Table S1** and **Figure S3**.

**SUPPLEMENTARY TABLES AND FIGURES**

**Supplementary Table S1. Parameter priors, sampling distributions, sources and posteriors to model the cohort of people receiving opioid agonist treatment (OAT) in New South Wales.**

**Supplementary Table S2. Initial population distribution and yearly new entries. (A)** Initial cohort population distribution by incarceration status. (**B)** Initial population distribution by OAT status. (**C)** Initial population distribution by OAT status.

**Supplementary Table S3. Calibration data.**

**Supplementary Table S4. Overdose death rate per year from 2000-2016 by years since first entry into OAT from the cohort data.**

**Supplementary Table S5. Calibrated OAT transition rates weighted by the proportion on buprenorphine and methadone, including +/-20% uncertainty to obtain a prior distribution.**

**Supplementary Table S6. Sensitivity analyses to evaluate the impact of OAT programs assuming (A) exponentially increasing overdose mortality rate and (B) no relapse.**

**Supplementary Table S7. Ancova analysis.**

**Supplementary Figure S1. Comparison of the calibrated exponential function (black line) to data (red line) on the retention of individuals on OAT for buprenorphine (A) and methadone recipients (B).**

**Supplementary Figure S2. Model validation against data not used for the calibration.**

**Supplementary Figure S3. Prior and posterior parameter distributions**.

**Supplementary Figure S4. Relative change of overdose and other cause deaths averted among the OAT cohort in NSW (A) through the full OAT program and the OAT program in prison compared to no OAT provision and (B) due to post-incarceration release, no increased risk during first month on OAT and no increased risk during first month off OAT from 2001-2020.**

**Supplementary Table S1. Parameter priors, sampling distributions, sources and posteriors to model the cohort of people receiving opioid agonist treatment (OAT) in New South Wales.** PWUO: people who use opioids. RR: relative risk. OD: overdose.

| **Variables** | **Priors** | **Distribution** | **Reference** | **Posteriors** |
| --- | --- | --- | --- | --- |
| **Mortality and opioid use cessation and relapse** |  |  |  |  |
| Mortality rate $\mu_{1}$ from overdose per year in 2001* | Min:0.001-Max:0.05 | Uniform | Wide, based on^16-13^ | 0.003 [95%CI: 0.002-0.005] |
| Mortality rate $\mu_{2}$from other causes among PWUO per year in 2001* | Min:0.001-Max:0.05 | Uniform |  | 0.009 [95%CI: 0.007-0.012] |
| Mortality rate from other causes among those no longer using opioids (“former PWUO”) per year in 2001* | Min:0.001-Max:0.05 | Uniform |  | 0.006 [95%CI: 0.004-0.009] |
| Long term opioid use cessation rate per year | Min:0.05-Max:0.1 | Uniform | ^5^ | 0.052 [95%CI: 0.05-0.056] |
| Opioid use relapse rate per year among those in long term cessation | Min:0.005-Max:0.03 | Uniform | ^6-10^.*for minority that stop for >5y | 0.025 [95%CI: 0.022-0.027] |
| **Incarceration patterns and effect on mortality** |  |  |  |  |
| Reincarceration rates among those incarcerated in the past month or past year per year** | Min:0.00-Max:3.00 | Uniform | Wide, uninformed | 0.649 [95%CI: 0.34-0.892] |
| Relative risk of incarceration among those never/not incarcerated in past year compared to incarcerated in past year | Min:0.00-Max:1.00 | Uniform | Assumed to be < than among incarcerated <1y | 0.097 [95%CI: 0.051-0.192] |
| Relative risk of reincarceration among former PWUO incarcerated in the past year compared to current PWUO incarcerated in the past year | Min:0.1-Max:0.8 | Uniform | Low bound based on rate among gen pop, high bound based on rate among those on OAT^15^ | 0.177 [95%CI: 0.121-0.24] |
| Relative risk of incarceration among former PWUO never/not incarcerated in the past year compared to incarcerated in past year | Min:0.00-Max:1.00 | Uniform | Assumed to be < than among incarcerated <1y | 0.04 [95%CI: 0.002-0.099] |
| Prison exit rate per year | Min:1.00-Max:5.79 | Uniform | OATS**^2^** | 1.283 [95%CI: 1.016-1.592] |
| RR OD mortality in prison compared to never/not incarcerated in past year | 0.055 [95%CI: 0.02-0.14] | Lognormal^†^ | ^3,4^ | 0.06 [95%CI: 0.05-0.08] |
| RR other cause mortality in prison compared to never/not incarcerated in past year) | 0.31 [95%CI: 0.19-0.47] | Lognormal^†^ | ^3^ | 0.423 [95%CI: 0.381-0.448] |
| RR OD mortality among those released in past month compared to never/not incarcerated in the past year | 3.70 [95%CI: 2.22-5.18] | Lognormal^†^ | ^4^ | 4.032 [95%CI: 3.732-4.358] |
| RR OD mortality released in past year compared to never/not incarcerated in the past year | 1.70 [95%CI: 1.02-2.38] | Lognormal^†^ | ^4^ | 1.161 [95%CI: 1.045-1.341] |
| RR other cause mortality among those released in the past month compared to never/not incarcerated in the past year | 1.40 [95%CI: 0.84-1.96] | Lognormal^†^ | ^4^ | 1.657 [95%CI: 1.558-1.814] |
| **OAT engagement and effect on mortality** |  |  |  |  |
| OAT re-enrolment out of prison per year in 2001*** | Min:0.01, Max:2.00 | Uniform | Wide, uninformed | 0.814 [95%CI: 0.716-0.932] |
| OAT re-enrolment in prison per year | Min:0.01, Max:2.00 | Uniform | Wide, uninformed | 0.825 [95%CI: 0.59-1.112] |
| Transition rate from short OAT to long OAT per year | Min: 1.38, Max:2.07 | Uniform | See section 1.1 | 1.486 [95%CI: 1.401-1.604] |
| Dropout rate from first month or short duration OAT per year | Min:1.77, Max:2.66 | Uniform | See section 1.1 | 2.204 [95%CI: 2.112-2.334] |
| Dropout rate from long OAT per year | Min:0.13, Max:0.20 | Uniform | See section 1.1 | 0.163 [95%CI: 0.149-0.171] |
| RR overdose mortality on OAT in the community (compared to off OAT in the community) | 0.22 [95%CI: 0.13-0.35] | Lognormal^†^ | ^12^ | 0.174 [95%CI: 0.147-0.203] |
| RR other cause mortality on OAT in the community (compared to off OAT in the community) | 0.57[95%CI: 0.34-0.80] | Lognormal^†^ | ^12^ | 0.374 [95%CI: 0.343-0.415] |
| RR overdose and other cause mortality in the first month on OAT (compared to rest of time on OAT) | 1.97 [95%CI: 0.93-4.00] | Lognormal^†^ | ^12^ | 2.294 [95%CI: 1.926-2.71] |
| RR overdose and other cause mortality in the first month off OAT (compared to rest of time off OAT) | 2.38 [95%CI: 1.53-3.75] | Lognormal^†^ | ^12^ | 1.782 [95%CI: 1.459-2.394] |
| RR overdose mortality on OAT in prison (compared to off OAT in prison) | 0.12 [95%CI: 0.06-0.26] | Lognormal^†^ | ^3,4,14^ | 0.173 [95%CI: 0.15-0.211] |
| RR other cause mortality on OAT in prison (compared to off OAT in prison) | 0.32 [95%CI: 0.15-0.63] | Lognormal^†^ | ^3,4,14^ | 0.466 [95%CI: 0.401-0.565] |
| RR of reincarceration on OAT (compared to off OAT) | 0.80 [95%CI: 0.71–0.90] | Lognormal^†^ | ^15^ | 0.818 [95%CI: 0.792-0.84] |

*Linear increase of mortality rate from overdose and other causes was assumed as follows:

- Mortality rate from overdose per year $\mu_{1}=\mu_{1}\times0.18\times(\tau-\tau_{start})$ with $\tau_{start}=2001$

- Mortality rate from other causes among PWUO per year $\mu_{2}=\mu_{2}\times0.03\times(\tau-\tau_{start})$

- Mortality rate from other causes among those no longer using opioids (“former PWUO”) per year $\mu_{3}=\mu_{3}\times0.03\times(\tau-\tau_{start})$

**RR= 1.2 applied before 2010.

***Linear increase in OAT enrolment in the community as follows: OAT enrolment in the community per year $\gamma=\gamma\times0.05\times(\tau-\tau_{start})$

# ^†^Priors with lognormal distribution were truncated to the lower and upper prior boundaries.

**Supplementary Table S2. Initial population distribution in 2001 and yearly new entries.**

1. **Initial cohort population distribution by incarceration status**

| Year | % in prison | % within 1 month post-release | % within >1-12 months post-release | % with no-past year incarceration | Initial cohort population size |
| --- | --- | --- | --- | --- | --- |
| 2001 | 8.5% | 1.2% | 12.9% | 77.4% | 21,086 |

1. **Initial population distribution by opioid agonist treatment (OAT) status**

| Year | % in 1^st^ month on OAT | % in short OAT episode | % in long OAT episode | % in 1^st^ month off OAT | % long period OFF OAT |
| --- | --- | --- | --- | --- | --- |
| 2001 | 13.2% | 19.7% | 32.9% | 10.2% | 23.9% |

1. **Yearly new opioid agonist treatment (OAT) cohort entries by incarceration status**

| **Year** | **Number entering OAT while in prison** | **Number entering OAT within 1 month post-release from prison** | **Number entering within >1-12 months post-release from prison** | **Number entering with no-past year custody** |
| --- | --- | --- | --- | --- |
| 2000 | 315 | 11 | 137 | 2370 |
| 2001 | 325 | 16 | 124 | 1461 |
| 2002 | 268 | 7 | 173 | 1749 |
| 2003 | 268 | 6 | 161 | 1523 |
| 2004 | 263 | 17 | 114 | 1433 |
| 2005 | 302 | 16 | 86 | 1214 |
| 2006 | 338 | 9 | 72 | 859 |
| 2007 | 344 | 4 | 52 | 919 |
| 2008 | 360 | 12 | 62 | 980 |
| 2009 | 381 | 15 | 55 | 1136 |
| 2010 | 362 | 10 | 58 | 1271 |
| 2011 | 103 | 21 | 64 | 1152 |
| 2012 | 82 | 37 | 77 | 1209 |
| 2013 | 130 | 45 | 101 | 1171 |
| 2014 | 133 | 48 | 97 | 1093 |
| 2015 | 86 | 58 | 93 | 1115 |
| 2016 | 106 | 105 | 114 | 1131 |
| 2017 | 94 | 161 | 147 | 1251 |
| 2018 | 148 | 43 | 130 | 996 |

**Supplementary Table S3. Calibration data.** OAT: opioid agonist treatment, NA: not available

| Year | Number of people in the cohort on 1st January | Total annual deaths | Number of people on OAT in prison on 1st January | Number of people on OAT outside prison on 1st January | Number of overdose deaths |
| --- | --- | --- | --- | --- | --- |
| 2001 | 21086 | 163 | 1055 | 12834 | 51 |
| 2002 | 22850 | 131 | 1066 | 12874 | 50 |
| 2003 | 24915 | 164 | 1233 | 13591 | 58 |
| 2004 | 26709 | 188 | 1385 | 13860 | 56 |
| 2005 | 28348 | 227 | 1329 | 13816 | 71 |
| 2006 | 29736 | 229 | 1436 | 14120 | 53 |
| 2007 | 30785 | 257 | 1524 | 13989 | 87 |
| 2008 | 31847 | 284 | 1501 | 14483 | 89 |
| 2009 | 32979 | 314 | 1804 | 14830 | 124 |
| 2010 | 34250 | 291 | 1753 | 15909 | 94 |
| 2011 | 34970 | 433 | 1522 | 16744 | 119 |
| 2012 | 35917 | 400 | 1100 | 16774 | 121 |
| 2013 | 36959 | 419 | 1099 | 17025 | 135 |
| 2014 | 38026 | 482 | 1151 | 17349 | 140 |
| 2015 | 38962 | 553 | 1137 | 17436 | 143 |
| 2016 | 39807 | 510 | 1226 | 17665 | 117 |
| 2017 | 40800 | 584 | 1151 | 18122 | NA |
| 2018 | 41923 | NA | 1133 | 18915 | NA |

*For years 2011 to 2018, a relative 10% was added to the total number of deaths and the number of overdose deaths to account for out of state deaths.

**Supplementary Table S3. Calibration data continued**

| Year | Proportion of death from overdose while on OAT (among all overdose deaths) | Proportion of death from other causes while on OAT (among all deaths from other causes) | Proportion ever incarcerated in the last year | Proportion currently incarcerated | Proportion overdose death first month post release (among all overdose deaths) | Proportion other causes death first month post release (among all overdose deaths) |
| --- | --- | --- | --- | --- | --- | --- |
| 2001 | 0.538 | 0.31 | 0.141 | 0.085 | 0.098 | 0.0268 |
| 2002 | 0.5 | 0.447 | 0.123 | 0.086 | 0.08 | 0.037 |
| 2003 | 0.212 | 0.347 | 0.12 | 0.084 | 0.0345 | 0.0094 |
| 2004 | 0.269 | 0.35 | 0.111 | 0.085 | 0.125 | 0.0076 |
| 2005 | 0.259 | 0.379 | 0.105 | 0.088 | 0.0563 | 0.0064 |
| 2006 | 0.275 | 0.307 | 0.102 | 0.085 | 0.0377 | 0.017 |
| 2007 | 0.21 | 0.32 | 0.095 | 0.089 | 0.046 | 0.0176 |
| 2008 | 0.228 | 0.287 | 0.092 | 0.083 | 0.0225 | 0.0103 |
| 2009 | 0.236 | 0.281 | 0.078 | 0.092 | 0.0484 | 0.0105 |
| 2010 | 0.303 | 0.305 | 0.081 | 0.08 | 0.0532 | 0.0203 |
| 2011 | 0.291 | 0.333 | 0.082 | 0.067 | 0.0648 | 0.0245 |
| 2012 | 0.246 | 0.298 | 0.083 | 0.059 | 0.0909 | 0.0079 |
| 2013 | 0.282 | 0.369 | 0.075 | 0.058 | 0.0569 | 0.0233 |
| 2014 | 0.302 | 0.347 | 0.072 | 0.059 | 0.0394 | 0.0129 |
| 2015 | 0.338 | 0.332 | 0.068 | 0.057 | 0.0846 | 0.0107 |
| 2016 | 0.336 | 0.321 | 0.068 | 0.06 | 0.1321 | 0.0098 |
| 2017 | NA | NA | 0.071 | 0.057 | NA | NA |
| 2018 | NA | NA | 0.067 | 0.055 | NA | NA |

**Supplementary Table S4. Overdose death rate per year from 2000-2016 by years since first entry into opioid agonist treatment (OAT) from the cohort data.**

| **Years since first entry into OAT** | **Year of death** | | | | | | | | | | | | | | | | |
| --- | --- | --- | --- | --- | --- | --- | --- | --- | --- | --- | --- | --- | --- | --- | --- | --- | --- |
|  | **2000** | **2001** | **2002** | **2003** | **2004** | **2005** | **2006** | **2007** | **2008** | **2009** | **2010** | **2011** | **2012** | **2013** | **2014** | **2015** | **2016** |
| **0-1** |  | 0.50% | 0.45% | 0.56% | 0.79% | 0.62% | 0.77% | 0.53% | 0.80% | 0.70% | 0.35% | 0.89% | 0.64% | 0.83% | 0.73% | 1.11% | 0.89% |
| **1-2** |  | . | 0.22% | 0.55% | 0.61% | 1.01% | 0.81% | 1.63% | 1.14% | 1.16% | 0.64% | 0.71% | 0.98% | 1.07% | 0.55% | 1.10% | 1.19% |
| **2-3** |  | . | . | 0.22% | 0.55% | 0.77% | 0.37% | 0.57% | 0.94% | 0.30% | 0.95% | 0.51% | 1.12% | 0.76% | 0.50% | 1.12% | 0.96% |
| **3-4** |  | . | . | . | 0.22% | 0.78% | 0.88% | 0.38% | 0.83% | 1.19% | 0.69% | 1.03% | 0.78% | 0.71% | 0.92% | 0.58% | 0.49% |
| **4-5** |  | . | . | . | . | 0.34% | 0.32% | 0.68% | 0.70% | 0.51% | 0.80% | 1.08% | 0.96% | 0.59% | 0.90% | 1.08% | 0.51% |
| **5-6** |  | . | . | . | . | . | 0.40% | 0.33% | 0.58% | 0.33% | 0.32% | 1.13% | 0.55% | 0.82% | 0.73% | 0.60% | 0.62% |
| **6-7** |  | . | . | . | . | . | . | 0.34% | 0.56% | 0.90% | 0.49% | 1.03% | 0.73% | 0.55% | 0.83% | 0.73% | 0.30% |
| **7-8** |  | . | . | . | . | . | . | . | 0.46% | 0.47% | 0.58% | 1.04% | 0.46% | 0.73% | 0.72% | 0.98% | 0.80% |
| **8-9** |  | . | . | . | . | . | . | . | . | 0.40% | 0.42% | 0.74% | 0.88% | 0.85% | 0.90% | 0.96% | 0.76% |
| **9-10** |  | . | . | . | . | . | . | . | . | . | 0.29% | 0.66% | 0.64% | 0.61% | 0.60% | 1.90% | 0.57% |
| **10-11** |  | . | . | . | . | . | . | . | . | . | . | 0.17% | 0.86% | 0.59% | 0.73% | 0.60% | 1.08% |
| **11-12** |  | . | . | . | . | . | . | . | . | . | . | . | 0.47% | 0.96% | 0.65% | 0.79% | 0.88% |
| **12-13** |  | . | . | . | . | . | . | . | . | . | . | . | . | 0.47% | 0.73% | 0.66% | 0.74% |
| **13-14** |  | . | . | . | . | . | . | . | . | . | . | . | . | . | 0.53% | 1.02% | 0.72% |
| **14-15** |  | . | . | . | . | . | . | . | . | . | . | . | . | . | . | 0.90% | 0.49% |
| **15-16** |  | . | . | . | . | . | . | . | . | . | . | . | . | . | . | . | 0.30% |

**Supplementary Table S5. Calibrated opioid agonist treatment (OAT) transition rates weighted by the proportion on buprenorphine and methadone, including +/-20% uncertainty to obtain a prior distribution.**

| **Parameters** | **Uniform priors (minimum-maximum)** |
| --- | --- |
| Transition rate from short OAT to long OAT per year ($1/a$) | 1.38-2.07 |
| Dropout rate from first month or short duration OAT per year $(\kappa)$ | 1.77-2.66 |
| Dropout rate from long OAT per year ($\pi)$ | 0.13-0.20 |

**Supplementary Table S6. Sensitivity analyses to evaluate the impact of OAT programs assuming (A) exponentially increasing overdose mortality rate and (B) no relapse.** A. Mortality rate from overdose per year $\mu_{1}=\mu_{1}\times exp(0.11\left( \tau-\tau_{start} \right))$ with $\tau_{start}=2001$

| **Alternative scenarios** |  | **Overdose deaths, 2001-2020**  Mean  [95%I] | **Change in overdose deaths compared to baseline with OAT**  Mean  [95%I] | **% overdose deaths averted, 2001-2020**  Mean  [95%I] | **Deaths from other causes, 2001-2020**  Mean [95%I] | **Change in other cause deaths compared to baseline with OAT**  Mean  [95%I] | **% other cause deaths averted, 2001-2020**  Mean  [95%I] | **Total deaths, 2001-2020**  Mean  [95%] | **Change in total deaths compared to baseline with OAT**  Mean  [95%I] | **% total deaths averted, 2001-2020**  Mean  [95%I] |
| --- | --- | --- | --- | --- | --- | --- | --- | --- | --- | --- |
| *A. Exponential increase overdose mortality rate* | *Baseline (with OAT)* | 2289 [95%I:1305-3412] | - |  | 5654 [95%I:4274-6827] | - |  | 7943 [95%I:6785-9079] | - |  |
|  | *No OAT* | 5151 [95%I:2981-7481] | 2862 [95%I:1612-4241] | 55.59 [95%I:49.42-59.57] | 7302 [95%I:5303-8885] | 1648 [95%I:929-2400] | 22.42 [95%I:15.58-29.44] | 12452 [95%I:10398-14525] | 4509 [95%I:3146-5797] | 36.06 [95%I:28.99-41.62] |
| *B. No relapse* | *Baseline (with OAT)* | 1931 [95%I:948-2891] | - |  | 4976 [95%I:3424-6560] | - |  | 6907 [95%I:5569-8313] | - |  |
|  | *No OAT* | 3919 [95%I:1909-5858] | 1988 [95%I:974-3044] | 50.68 [95%I:46.46-55.15] | 6308 [95%I:4383-8303] | 1332 [95%I:828-1886] | 21.07 [95%I:15.99-25.86] | 10227 [95%I:8358-12187] | 3320 [95%I:2403-4318] | 32.42 [95%I:26.11-38.49] |

**Supplementary Table S7. Ancova analysis.** OAT: opioid agonist treatment, RR: relative risk

| **Variable** | **% of Sum of Squares** |
| --- | --- |
| RR overdose mortality on OAT in the community (compared to off OAT in the community) | 56.3 |
| RR other cause mortality on OAT in the community (compared to off OAT in the community) | 20.4 |
| RR overdose and other cause mortality in the first month off OAT (compared to rest of time off OAT) | 9.2 |
| RR overdose and other cause mortality in the first month on OAT (compared to rest of time on OAT) | 5.9 |
| Long term opioid use cessation rate per year | 4.7 |
| RR OD mortality in prison compared to never/not incarcerated in past year | 1.2 |
| Opioid use relapse rate per year among those in long term cessation | 1.1 |
| Relative risk of incarceration among those never/not incarcerated in past year compared to incarcerated in past year | 0.3 |
| RR OD mortality among those released in past month compared to never/not incarcerated in the past year | 0.3 |
| Prison exit rate per year | 0.3 |
| RR overdose mortality on OAT in prison (compared to off OAT in prison) | 0.1 |
| Reincarceration rates among those incarcerated in the past month or past year per year | 0.1 |
| RR other cause mortality on OAT in prison (compared to off OAT in prison) | 0.1 |
| RR other cause mortality among those released in the past month compared to never/not incarcerated in the past year | 0 |
| RR of reincarceration on OAT (compared to off OAT) | 0 |
| RR OD mortality released in past year compared to never/not incarcerated in the past year | 0 |
| RR other cause mortality in prison compared to never/not incarcerated in past year) | 0 |

**Supplementary Figure S1. Comparison of the calibrated exponential function (black line) to data (red line) on the retention of individuals on opioid agonist treatment for buprenorphine (A) and methadone recipients (B).**

A.


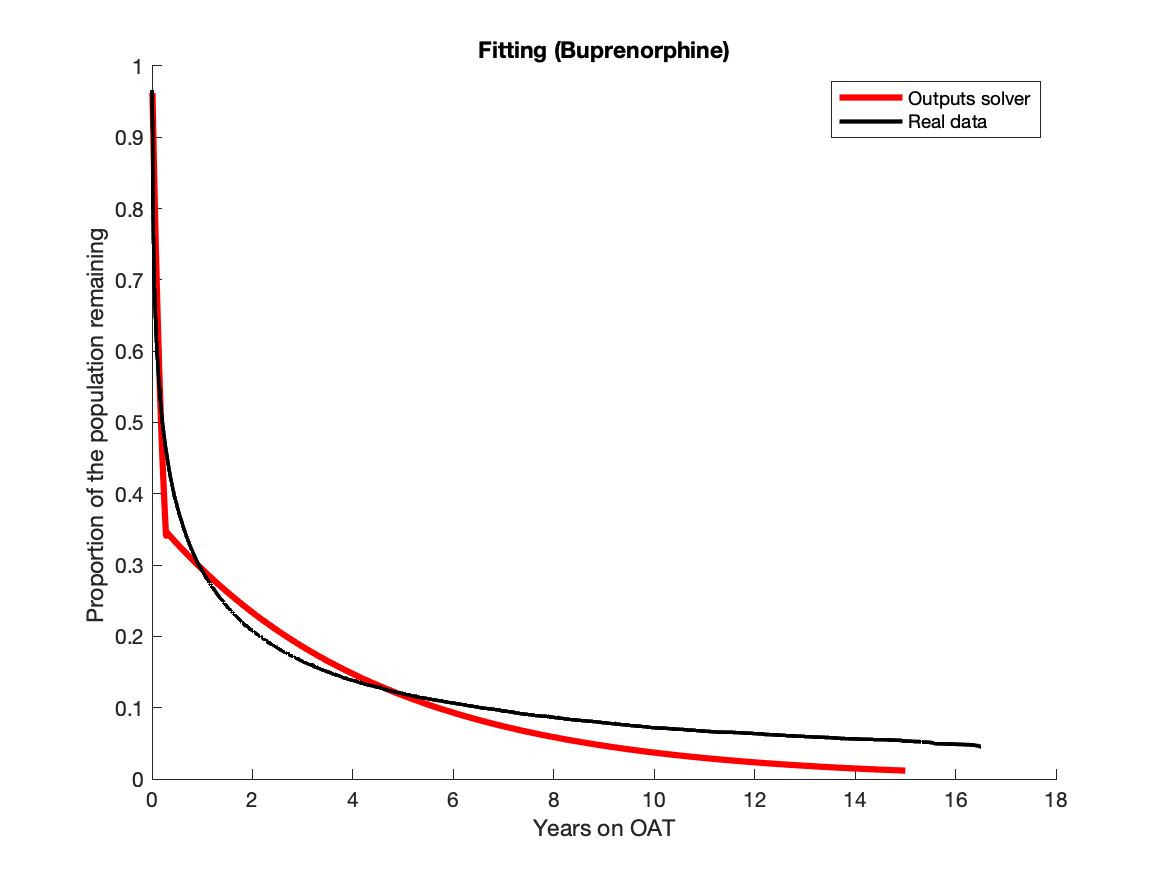


B.


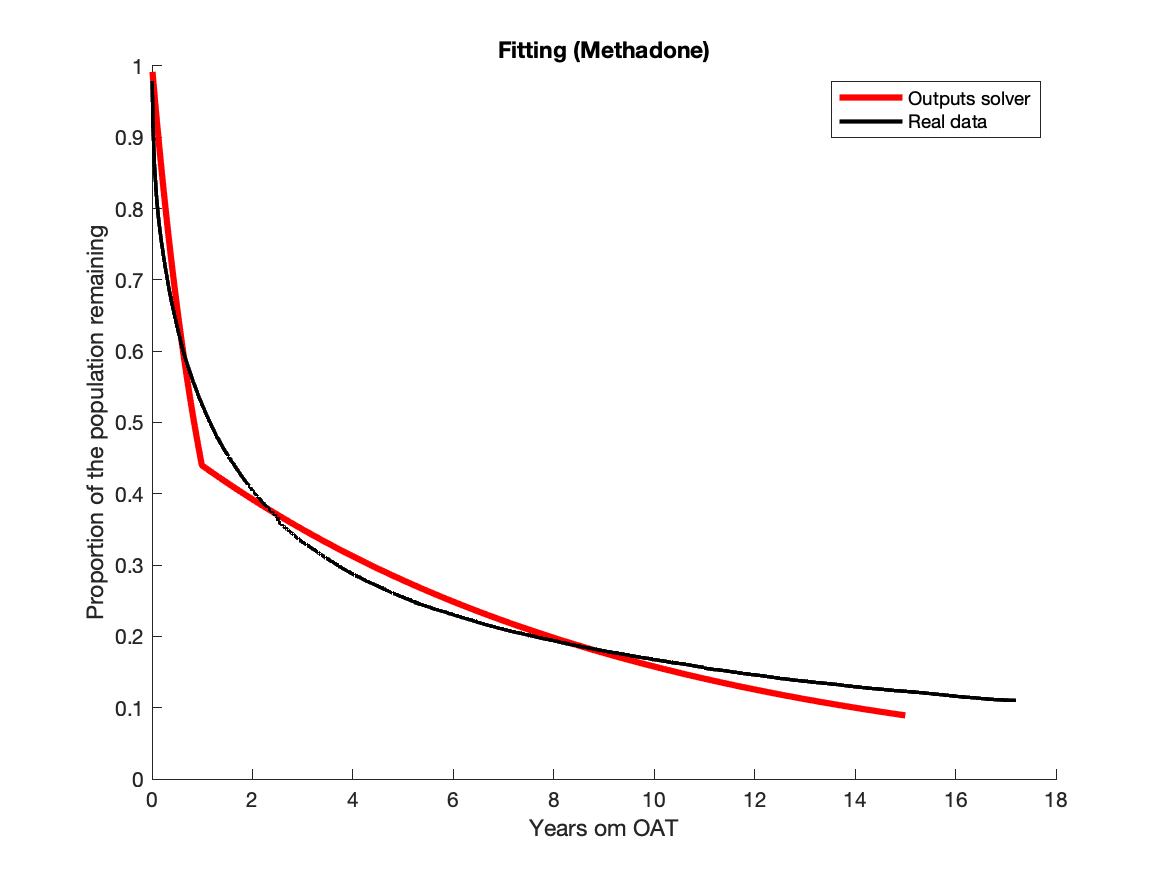


Short and long-term transition rates calculated overall by weighting by the proportion of individuals on methadone and buprenorphine (54% and 46% respectively)^17^ .

**Supplementary Figure S2. Model validation against data not used for the calibration.** OD: overdose

**
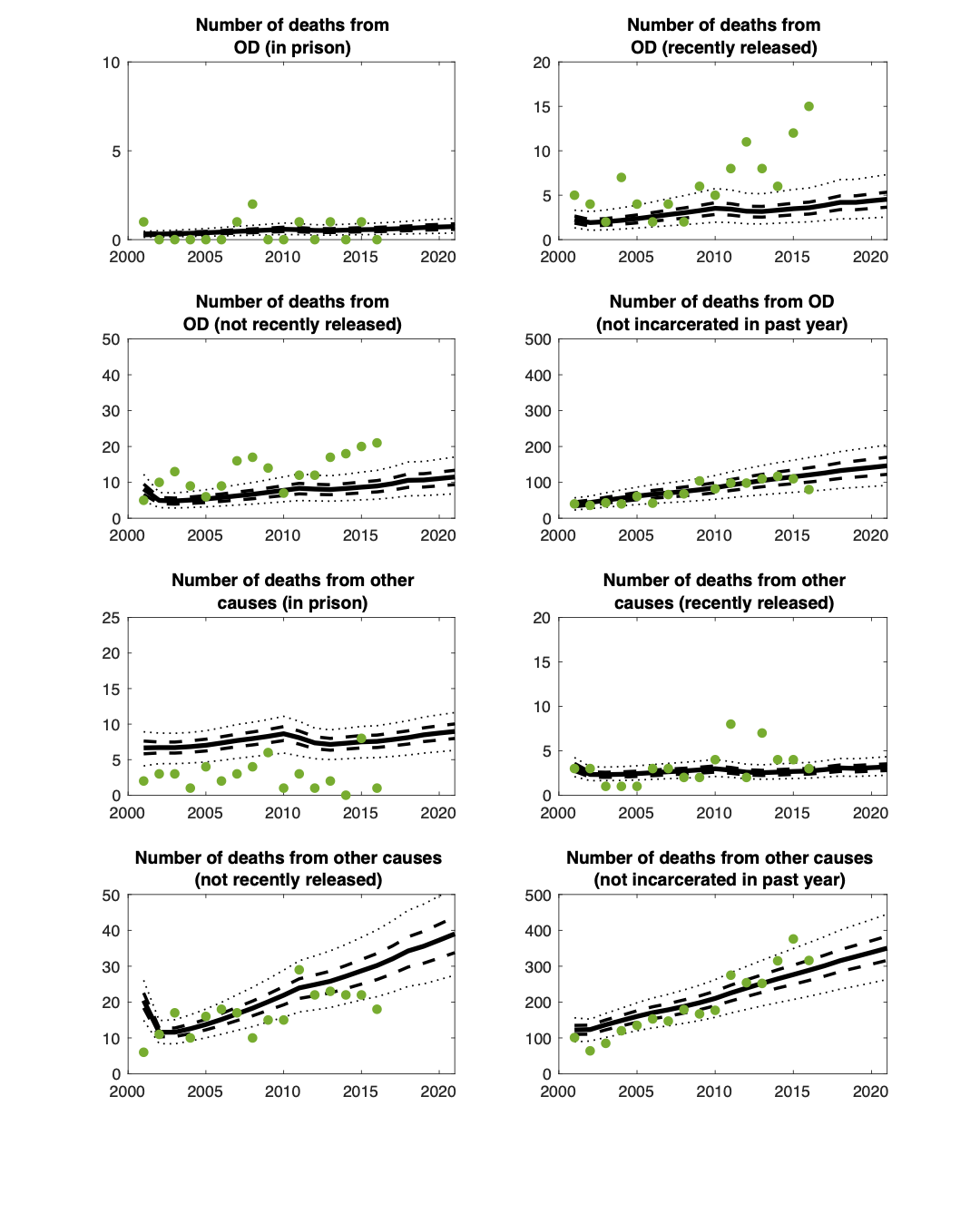
**

# Supplementary Figure S3. Prior and posterior parameter distributions. Red area denotes the prior distribution and vertical dotted lines show the lower and upper limits of prior values. Posterior distributions are depicted by gray bars. Priors with lognormal distribution were truncated to the lower and upper prior boundaries. OAT: opioid agonist treatment.

**Supplementary Figure S4. Relative change of overdose and other cause deaths averted among the OAT cohort in NSW (A) through the full OAT program and the OAT program in prison compared to no OAT provision and (B) due to post-incarceration release, no increased risk during first month on OAT and no increased risk during first month off OAT from 2001-2020.** OAT: opioid agonist treatment

**(A)**

**
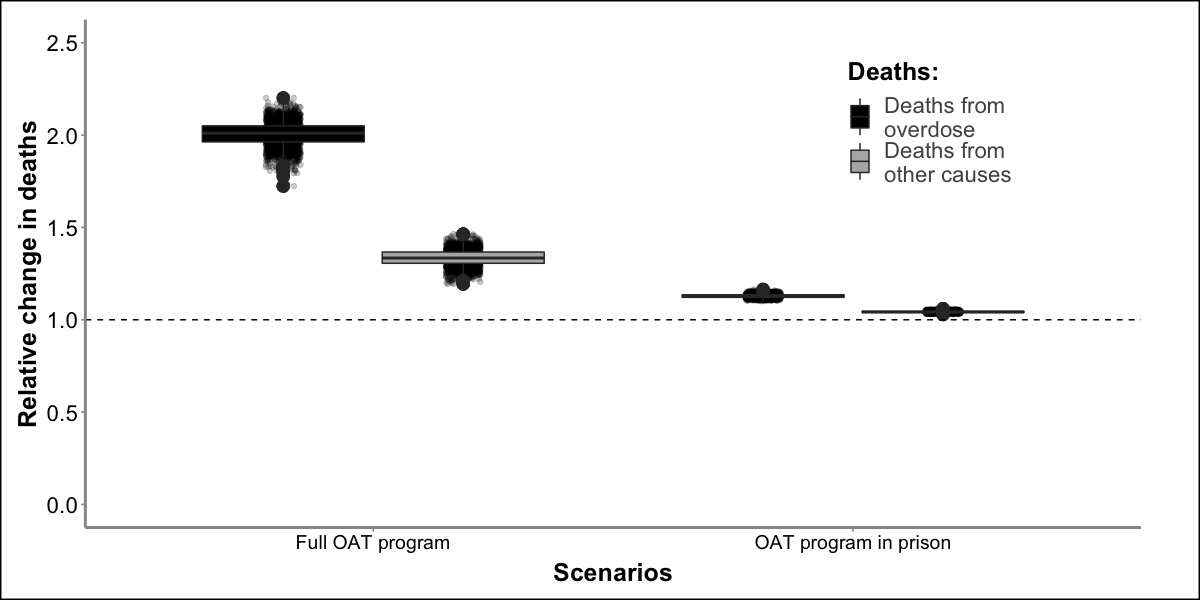
**

**(B)**

REFERENCES

1 Centre for Health Record Linkage. *Electronic Recording and Reporting of Controlled Drugs (ERRCD) database*, <<https://www.cherel.org.au/data-dictionaries#section24>> (2021).

2 Larney, S. *et al.* Using routinely collected data to understand and predict adverse outcomes in opioid agonist treatment: Protocol for the Opioid Agonist Treatment Safety (OATS) Study. *BMJ open* **8**, e025204, doi:10.1136/bmjopen-2018-025204 (2018).

3 Larney, S. *et al.* Opioid substitution therapy as a strategy to reduce deaths in prison: retrospective cohort study. *BMJ open* **4**, e004666, doi:10.1136/bmjopen-2013-004666 (2014).

4 Degenhardt, L. *et al.* The impact of opioid substitution therapy on mortality post-release from prison: retrospective data linkage study. *Addiction (Abingdon, England)* **109**, 1306-1317, doi:10.1111/add.12536 (2014).

5 Calabria, B. *et al.* Systematic review of prospective studies investigating "remission" from amphetamine, cannabis, cocaine or opioid dependence. *Addict Behav* **35**, 741-749, doi:10.1016/j.addbeh.2010.03.019 (2010).

6 Termorshuizen, F. *et al.* Prediction of relapse to frequent heroin use and the role of methadone prescription: An analysis of the Amsterdam Cohort Study among drug users. *Drug and Alcohol Dependence* **79**, 231-240, doi:<https://doi.org/10.1016/j.drugalcdep.2005.01.013> (2005).

7 Shah, N. G., Galai, N., Celentano, D. D., Vlahov, D. & Strathdee, S. A. Longitudinal predictors of injection cessation and subsequent relapse among a cohort of injection drug users in Baltimore, MD, 1988-2000. *Drug Alcohol Depend* **83**, 147-156, doi:10.1016/j.drugalcdep.2005.11.007 (2006).

8 Mehta, S. H. *et al.* Factors associated with injection cessation, relapse and initiation in a community-based cohort of injection drug users in Chennai, India. *Addiction (Abingdon, England)* **107**, 349-358, doi:10.1111/j.1360-0443.2011.03602.x (2012).

9 Hser, Y. I., Evans, E., Grella, C., Ling, W. & Anglin, D. Long-term course of opioid addiction. *Harv Rev Psychiatry* **23**, 76-89, doi:10.1097/hrp.0000000000000052 (2015).

10 Goldman-Hasbun, J., Nosova, E., Kerr, T., Wood, E. & DeBeck, K. Homelessness and incarceration associated with relapse into stimulant and opioid use among youth who are street-involved in Vancouver, Canada. *Drug and alcohol review* **38**, 428-434, doi:10.1111/dar.12921 (2019).

11 Mukandavire, C. *et al.* Impact of opioid substitution therapy on the HIV prevention benefit of antiretroviral therapy for people who inject drugs. *Aids* **31**, 1181-1190, doi:10.1097/qad.0000000000001458 (2017).

12 Sordo, L. *et al.* Mortality risk during and after opioid substitution treatment: systematic review and meta-analysis of cohort studies. *BMJ* **357**, j1550, doi:10.1136/bmj.j1550 (2017).

13 Degenhardt, L. *et al.* Global patterns of opioid use and dependence: harms to populations, interventions, and future action. *Lancet* **394**, 1560-1579, doi:10.1016/s0140-6736(19)32229-9 (2019).

14 Degenhardt, L. *et al.* Mortality among clients of a state-wide opioid pharmacotherapy program over 20 years: risk factors and lives saved. *Drug Alcohol Depend* **105**, 9-15, doi:10.1016/j.drugalcdep.2009.05.021 (2009).

15 Larney, S., Toson, B., Burns, L. & Dolan, K. Effect of prison-based opioid substitution treatment and post-release retention in treatment on risk of re-incarceration. *Addiction (Abingdon, England)* **107**, 372-380, doi:10.1111/j.1360-0443.2011.03618.x (2012).

16 Larney, S. *et al.* All-Cause and Cause-Specific Mortality Among People Using Extramedical Opioids: A Systematic Review and Meta-analysis. *JAMA Psychiatry* **77**, 493-502, doi:10.1001/jamapsychiatry.2019.4170 (2020).

17 Burns, L. *et al.* A longitudinal comparison of retention in buprenorphine and methadone treatment for opioid dependence in New South Wales, Australia. *Addiction (Abingdon, England)* **110**, 646-655, doi:10.1111/add.12834 (2015).
